# Bilateral scleritis and sclerokeratitis associated with IgA nephropathy

**DOI:** 10.1007/s12348-012-0069-7

**Published:** 2012-03-13

**Authors:** Manuel Garza-Leon, Diana Flores, Gabriela Alarcón-Galván, Concepción Sánchez-Martínez

**Affiliations:** 1 Instituto para Preservación de la Visión, Destellos de Luz I.B.P, Ruperto Martínez # 1317 Pte., Col. Centro, Monterrey, Nuevo León 64000 Mexico; 2 Department of Rheumatology, Hospital Universitario Dr. Jose E. Gonzalez, Universidad Autónoma de Nuevo León, Monterrey, Mexico; 3 Department of Pathology, Hospital Universitario Dr. Jose E. Gonzalez, Universidad Autónoma de Nuevo León, Monterrey, Mexico; 4 Kidney Unit, Hospital Universitario Dr. Jose E. Gonzalez, Universidad Autónoma de Nuevo León, Monterrey, Mexico

## Abstract

**Purpose:**

The purpose of this study is to report a case of bilateral nodular scleritis in a patient with final diagnosis of IgA nephropathy.

**Methods:**

This is an observational case report.

**Results:**

A male patient, 42 years old, presented with a bilateral nodular scleritis and OD sclerokeratitis. He had a previous history of acute otitis media and developed posterior renal failure and arterial hypertension. Clinical and systemic findings suggest Wegener's granulomatosis. A kidney biopsy was performed, and immunoflourescence findings demonstrated granular deposits of IgA in a mesangial pattern confirming the diagnosis of IgA nephropathy

**Conclusions:**

IgA nephropathy should be a differential diagnosis in patients with scleritis and nephropathy.

## Introduction

Scleritis may occur as an isolated phenomenon or as a manifestation of a variety of rheumatic disorders, infectious diseases, and metabolic disorders [1]. Scleritis is associated with systemic disease in approximately 50 % of cases [2]; however, it is not frequent in IgA nephropathy (IgAN).

IgAN, first described by Berger in 1969 [3], is one of the most common primary glomerulopathies worldwide [4]. The disease is diagnosed by renal biopsy using histopathologic and immunoflourescence tests. The hallmark of IgAN is macroscopic hematuria that usually occurs with or immediately after an upper airway infection [5]. Other characteristic manifestations are: painless episodic hematuria, proteinuria, hypertension, and renal impairment. IgA nephropathy was previously thought to be a benign disease with a 10-year renal survival rate greater than 80 %, but emerging data show a different scenario. Most patients develop a progressive decline in renal function, and about 40 % present end-stage renal failure [6]. Although IgAN is clinically limited to the kidneys in most cases, there are associations with other conditions, particularly with immune and inflammatory diseases, such as ankylosing spondylitis, rheumatoid arthritis, Reiter syndrome and Behcet's disease, celiac disease, alcoholic and non-alcoholic liver disease, hepatic schistosomiasis, pulmonary sarcoidosis, and dermatitis herpetiformis [7].

Ocular involvement in patients with IgAN is infrequent, and the most common association is with uveitis [8–11]. Other ophthalmic manifestations that are present with IgAN are episcleritis [12, 13], scleritis [14, 15], retinal vasculitis [8], serous retinal detachment [16], ciliochoroidal effusion [17], and Vogt–Koyanagi–Harada syndrome. [18]

The ocular involvement could be related to immunopathogenic alterations probably associated with the dysregulation of innate immunity and complement system activation [5] and also related with systemic complications such as renal failure and hypertension [19]. We present a case of IgAN associated with bilateral nodular scleritis, first diagnosed as Wegener's granulomatosis, with IgAN confirmed after renal biopsy.

## Case report

A 42-year-old man was referred due to a 1-month history of redness, ocular pain, and blurred vision in both eyes. His past medical history included acute otitis media complicated with bacterial pneumonia 1 year previous. He later presented with renal failure and arterial hypertension. He also had had diabetes for the past 10 years.

**Fig. 1:**
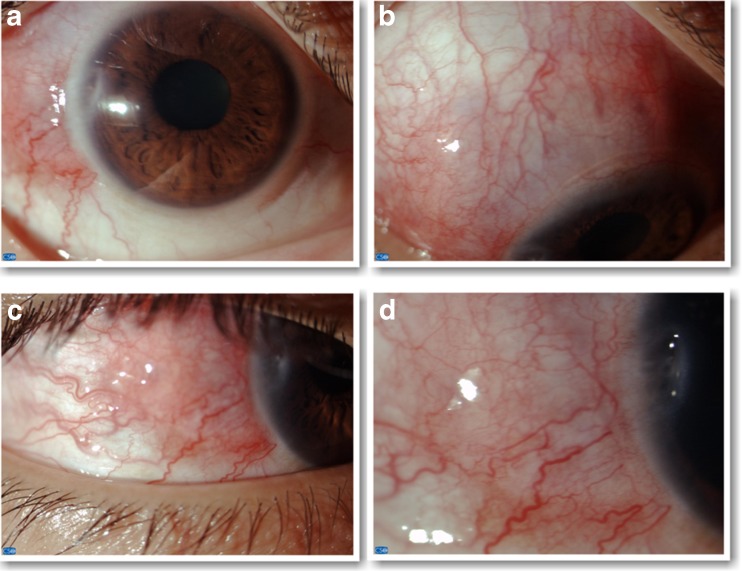
Right eye. **a** Slit-lamp photograph with low magnification showing an inflamed, elevated, immobile, scleral nodule in the temporal part of the sclera. **b** Slit-lamp photograph with ×16 magnification showing diffuse superior scleral inflammation. **c** Slit-lamp photograph with ×16 magnification showing the temporal sclera nodule. **d** Slit-lamp photograph with ×16 magnification showing thinning of the cornea at IX meridian

**Fig. 2:**
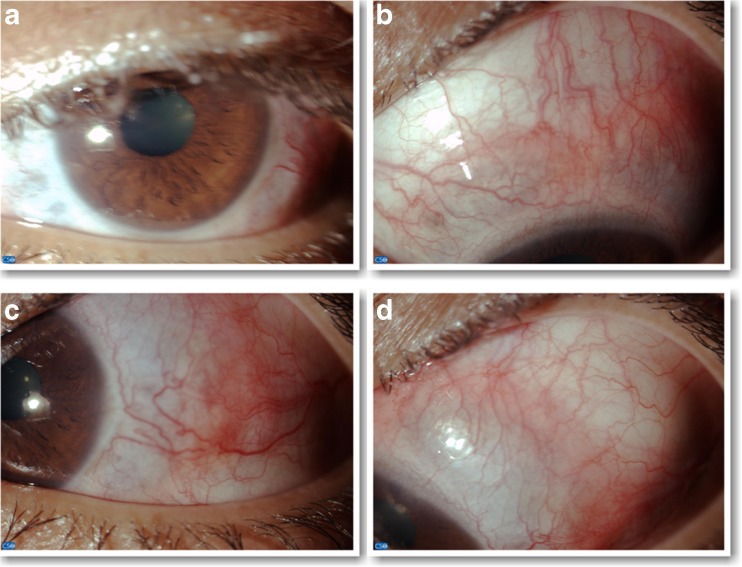
Left eye. **a** Slit-lamp photograph with low magnification showing an inflamed, elevated, immobile, scleral nodule in the temporal part of the sclera. **b** Slit-lamp photograph with ×16 magnification showing a superior nodular scleritis. **c** Slit-lamp photograph with ×16 magnification showing the temporal sclera nodule. **d** Slit-lamp photograph with ×16 magnification demonstrating purple coloration of the sclera

Ophthalmic examination revealed a visual acuity of 20/50 OD and 20/80 OS. There was an immovable, tender, inflamed scleral nodule on the temporal side of the sclera of the right eye associated with diffuse superior scleral inflammation and thinning of the cornea at IX meridian (Fig. 1). In the left eye, there were two nodules in the temporal and superior sclera associated with diffuse scleral inflammation (Fig. 2). The anterior chamber was deep with 0.5+ cells (Standardization of Uveitis Nomenclature Working Group [20]) in the OD and no cells in OS. The rest of the examination was normal. The diagnosis of bilateral nodular scleritis and sclerokeratitis in OD was made; because of the clinical and systemic findings, Wegener's granulomatosis was considered, and high-dose steroid treatment was begun after infectious causes were ruled out.

Laboratory tests included a complete blood count, an erythrocyte sedimentation rate, antinuclear antibodies, liver enzymes, c-ANCA and p-ANCA, CRP, urinalysis, chest x-ray, thoracic CT, VDRL, a fluorescent treponemal antibody absorption test, and a purified protein derivative skin test for tuberculosis. Serum urea nitrogen was 94 mg/dl and serum creatinine was 2.7 mg/dl; antinuclear antibodies were found to be 1:20 and were considered negative. The rest of the laboratory assessment was negative.

**Fig. 3:**
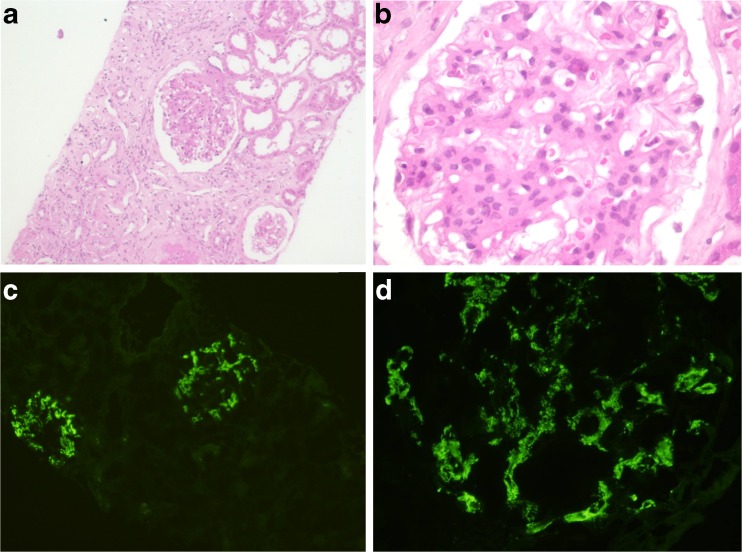
**a** H&E, ×10. Interstitial fibrosis and tubular atrophy. **b** H&E, ×40. Mesangial hyperplasia. **c** IF-IgA, ×5. Positive glomeruli and negative interstice. **d** IF-IgA, ×40. Mesangial and granular pattern

Kidney biopsy was performed because both antineutrophil cytoplasmic antibodies (c- and p-ANCA) and antibodies to proteinase-3 and myeloperoxidase were negative, and thoracic CT abnormalities were not found. Pathologic findings consisted of an adequate kidney biopsy (28 glomeruli) with 53 % global glomerulosclerosis, moderate tubular atrophy, and moderate interstitial fibrosis. The rest of the glomeruli showed diffuse proliferative mesangial hypercellularity with an increase of the mesangial matrix. The biopsy did not show endocapillary hypercellularity, crescents, segmental sclerosis, glomerulitis, vasculitis, or necrosis. Glomerular basal membrane thickness was normal. Immunofluorescence demonstrated granular deposits of IgA in a mesangial pattern with strong intensity (4 out of 4 points). Glomerular staining for IgG, IgM, lambda with less intensity (2 out of 4 points), and kappa predominance (3 out of 4 points) were also found. Others markers such as C1q, C3c, C4c, fibrin, and albumin were negative (Fig. 3).

After one monthly pulse of IV cyclophosphamide therapy, the scleral nodules resolved completely, and there was development of mild scleral thinning. At 2 years of follow-up with azathioprine, there are no recurrences, and mild renal failure is stable.

## Discussion

IgA nephropathy is the most common form of primary glomerular disease in the developed world [4]. Of the primary glomerular diseases, IgA nephropathy and isolated mesangial C3 deposits have been associated with scleritis and episcleritis [12, 14].

In IgAN, the deposits of IgA are frequently associated with complement components. The alternative pathway components C3 and properdin, and the membrane attack complex, are generally found in the mesangial deposits in IgAN, while the classical pathway components C1q and C4 are usually absent [21].

The role of circulating immune complexes in the pathogenesis of IgAN is controversial. Hall and coworkers [22] found IgA circulating immune complexes in patients with early stages of the disease, and Sirbat and associates [12] found numerous dimeric-IgA-secreting cells in an episcleral biopsy obtained from a female patient with IgAN and frequent episodes of episcleritis.

Nomoto and coworkers [14] followed 113 patients with various types of primary glomerular diseases for 1–33 months and verified that, of the patients studied, six exhibited scleritis. All of these six patients with scleritis were identified as having IgAN. Importantly, none of the patients other than those with IgAN had scleritis during the study period.

This case fits the systemic patterns of IgAN previously reported [5, 23]. Our patient began with an acute upper airway infection that often precedes clinical exacerbation of IgA nephropathy. Arterial hypertension can be present in 29 to 36 % of these patients at the beginning of the disease.

The most important differential diagnosis considered was Wegener's granulomatosis. The negative laboratory results and the normal chest x-ray and thoracic CT suggested a kidney biopsy to confirm the diagnosis.

In patients with scleritis and renal and urine abnormalities associated with a history of upper airway infection, IgAN should be considered and investigated. We hypothesize that the abnormalities of the IgA immune system, similar to those of IgA nephropathy, may be involved in the development of scleritis.

## Conclusion

In summary, we present a case of scleritis and sclerokeratitis associated with IgA nephropathy in a patient with a history of an acute upper airway infection and later development of nephropathy and hypertension. Negative imaging and laboratory findings suggested the need of a kidney biopsy for the differential diagnosis between Wegener's granulomatosis and IgA nephropathy.
